# Implicit and Explicit Motivational Tendencies to Faces Varying in Trustworthiness and Dominance in Men

**DOI:** 10.3389/fnbeh.2018.00008

**Published:** 2018-01-23

**Authors:** Sina Radke, Theresa Kalt, Lisa Wagels, Birgit Derntl

**Affiliations:** ^1^Department of Psychiatry, Psychotherapy and Psychosomatics, Medical Faculty, RWTH Aachen University, Aachen, Germany; ^2^JARA - BRAIN Institute Brain Structure-Function Relationships: Decoding the Human Brain at Systemic Levels, RWTH Aachen University, Aachen, Germany; ^3^Institute of Neuroscience and Medicine (INM-10), Research Center Jülich, Jülich, Germany; ^4^Department of Psychiatry and Psychotherapy, Medical School, University of Tübingen, Tübingen, Germany; ^5^Werner Reichardt Center for Integrative Neuroscience, University of Tübingen, Tübingen, Germany; ^6^LEAD Graduate School, University of Tübingen, Tübingen, Germany

**Keywords:** approach-avoidance, trustworthiness, dominance, testosterone, 2D:4D

## Abstract

Motivational tendencies to happy and angry faces are well-established, e.g., in the form of aggression. Approach-avoidance reactions are not only elicited by emotional expressions, but also linked to the evaluation of stable, social characteristics of faces. Grounded in the two fundamental dimensions of face-based evaluations proposed by Oosterhof and Todorov (2008), the current study tested whether emotionally neutral faces varying in trustworthiness and dominance potentiate approach-avoidance in 50 healthy male participants. Given that evaluations of social traits are influenced by testosterone, we further tested for associations of approach-avoidance tendencies with endogenous and prenatal indicators of testosterone. Computer-generated faces signaling high and low trustworthiness and dominance were used to elicit motivational reactions in three approach-avoidance tasks, i.e., one implicit and one explicit joystick-based paradigm, and an additional rating task. When participants rated their behavioral tendencies, highly trustworthy faces evoked approach, and highly dominant faces evoked avoidance. This pattern, however, did not translate to faster initiation times of corresponding approach-avoidance movements. Instead, the joystick tasks revealed general effects, such as faster reactions to faces signaling high trustworthiness or high dominance. These findings partially support the framework of Oosterhof and Todorov (2008) in guiding approach-avoidance decisions, but not behavioral tendencies. Contrary to our expectations, neither endogenous nor prenatal indicators of testosterone were associated with motivational tendencies. Future studies should investigate the contexts in which testosterone influences social motivation.

## Introduction

Approach and avoidance motivation are fundamental in regulating behavior. While avoiding potentially harmful stimuli ensures survival, approaching potentially rewarding stimuli facilitates thriving (Elliot, 2008). In research with human participants, these motivational tendencies can be reliably quantified by approach-avoidance tasks, in which participants pull a joystick toward (approach) or push it away from their body (avoidance). For emotional facial expressions, healthy individuals are typically faster to initiate approach movements to happy faces and to initiate avoidance movements to angry faces than vice versa (Rotteveel and Phaf, 2004).

Not only emotional expressions, but also expressions conveying socially relevant information may serve as signals whether to approach or avoid a person (Oosterhof and Todorov, 2008). Structural facial features, such as the distance between the eyes and the eyebrows or the facial width-to-height ratio, bias holistic inferences on social traits and behavioral patterns, e.g., aggression (Carré et al., 2009a; Shasteen et al., 2015). Thus, it is not surprising that common mechanisms underlie the evaluation of emotional expressions, e.g., anger, and of social traits, e.g., trustworthiness (Todorov and Engell, 2008; Engell et al., 2010). In fact, untrustworthy faces are likely to be perceived as angry or threatening, signaling avoidance behavior (Oosterhof and Todorov, 2008). The data-driven model by Oosterhof and Todorov (2008) has identified two underlying orthogonal dimensions of face evaluation which account for more than 80% of the variance: The first dimension can be approximated via trustworthiness judgments, i.e., the intention of a person to cause harm. The second dimension can be interpreted as dominance, i.e., the ability of a person to inflict harm. This model has been extensively validated (Oosterhof and Todorov, 2008; Dotsch and Todorov, 2012; Todorov et al., 2013), and provides the opportunity to manipulate the stable facial cues associated with trustworthiness and dominance, respectively, while keeping other visual features constant. Although faces with mild variations in the two dimensions are rated as emotionally neutral, they have been proposed to elicit motivational tendencies (Oosterhof and Todorov, 2008).

Along these lines, slower approach movements toward untrustworthy, compared to trustworthy faces, were obtained in a joystick-based approach-avoidance task (Slepian et al., 2012). Here, participants had to react to faces and houses, without being aware of the faces varying in trustworthiness. As approach-avoidance tendencies are most consistently found when expressions are explicitly evaluated (e.g., “if you see a happy face, pull the joystick toward yourself”) and to a lesser extent with implicit task instructions (Phaf et al., 2014), the current study set out to test in how far implicit and explicit evaluations influence behavioral tendencies towards faces varying in social traits. In light of the mixed findings, we investigated several “levels” of approach-avoidance tendencies to stimuli signaling varying degrees of trustworthiness and dominance. Although mainly natural faces have been investigated in approach-avoidance tasks, we used the computerized faces from Oosterhof and Todorov (2008) to adhere to the proposed framework, and to enable control of other features of the face (e.g., symmetry, hair) that influence trustworthiness judgments (e.g., Bakmazian, 2014). Rinck et al. (2010) also emphasized the advantage of immersive virtual environments in perfect experimental control over facial expressions and observed similar approach-avoidance behavior as in real-life settings. Inferences about trustworthiness from facial appearance are elicited by both natural and computerized faces (Klapper et al., 2016), so that, putting the proposition of Oosterhof and Todorov (2008) to test, we expected highly trustworthy faces to elicit approach and highly dominant faces to elicit avoidance.

Interestingly, evaluations of these social traits are influenced by the steroid hormone testosterone. Administration of testosterone decreased ratings of facial trustworthiness in females (Bos et al., 2010), but not in males (Bird et al., 2017). For endogenous testosterone, sex- and context-dependent effects were observed as a rise in testosterone levels after a competitive interaction predicted decreased trust ratings in men (but not in women; Carré et al., 2014). Exogenous testosterone did not alter males’ perceptions of dominance in emotionally neutral faces (Bird et al., 2017), but increased their self-perceived dominance (Welling et al., 2016).

Changes in the perception of social signals may influence engagement in motivational behavior such as aggression. For social threat conveyed by emotional valence, testosterone has been shown to bias behavior toward the approach of social threat, i.e., angry faces (Enter et al., 2014, 2016), subserved by increased amygdala activation (Radke et al., 2015b). As testosterone also increased amygdala reactivity to untrustworthy faces (Bos et al., 2012), behavioral approach toward faces signaling threat via the intention or the ability to cause harm, i.e., untrustworthy or dominant faces, may be similarly influenced by endogenous testosterone. In addition, these activational effects may be facilitated by testosterone’s impact on brain organization during prenatal development (Sisk and Zehr, 2005). The second-to-fourth digit ratio (2D:4D), a putative index of prenatal testosterone exposure (Zheng and Cohn, 2011), has been related to aggression and dominance (Turanovic et al., 2017). Therefore, we also tested for associations of approach-avoidance tendencies with endogenous and prenatal indicators of testosterone. In order to rely on a homogenous population, only males were included in this initial study.

## Materials and Methods

### Ethics Statement

This study was carried out in accordance with the recommendations of the World Medical Association Declaration of Helsinki with written informed consent from all subjects. The protocol was approved by the local ethics committee at the Medical Faculty of RWTH Aachen University.

### Participants and Procedure

The sample consisted of 50 healthy young men (*M*_age_ = 25.1 years, range = 18–33), recruited from the local university and via personal networks. Using an in-house checklist, participants were screened for severe somatic, endocrine and psychiatric disorders. Additional exclusion criteria were participation in a pharmacological study within the last month, use of medication, hormones or illegal substances, and smoking more than five cigarettes per day. Participants were asked to abstain from eating and drinking (except water) 3 h before the experimental session as saliva samples were obtained to determine testosterone concentration. All sessions took place between 2 pm and 5 pm, to control for diurnal hormonal variation. Sample size was based on previous research in this field (Rotteveel and Phaf, 2004; Slepian et al., 2012; Radke et al., 2013, 2014).

### Stimulus and Response Materials

Stimuli consisted of computer-generated faces, which were originally developed with FaceGen (Singular Inversions, Toronto, ON Canada) by Oosterhof and Todorov (2008), applying a 2D statistical model of face evaluation. This model was developed in a multi-step procedure, with, in short, first obtaining trait ratings of faces, then applying a principal component analysis to these ratings which reduced them to the dimensions of trustworthiness and dominance, and cross-validating the model with ratings on 300 computer-generated faces (Oosterhof and Todorov, 2008; Todorov et al., 2013). This database was chosen to enable a highly controlled manipulation of variations in facial features. In particular, specific structural facial features have been associated with the two dimensions, such as a larger distance between eyes and eyebrows with trustworthiness, and masculine features, e.g., a larger facial width-to-height ratio, with dominance. Keeping other features constant, and controlling for non-facial attributes, e.g., hair, which may influence evaluations of social traits (Bakmazian, 2014; Kalogiannidou and Peters, 2015), any differences in response should be caused by the manipulation in facial characteristics along the specific dimension.

Pictures from 25 different models were selected from the “fi” face set, all depicting Caucasian males without hair or facial hair in a front view. For each model, four pictures were used, i.e., a high and low expression of each feature of interest (trustworthiness, dominance), corresponding to the −2SD and +2SD version of each feature (out of seven available versions, ranging from −3SD to +3SD). This procedure resulted in a set of 100 experimental stimuli (during practice trials, different pictures, i.e., from the “nexus” set, were used).

**Figure 1:**
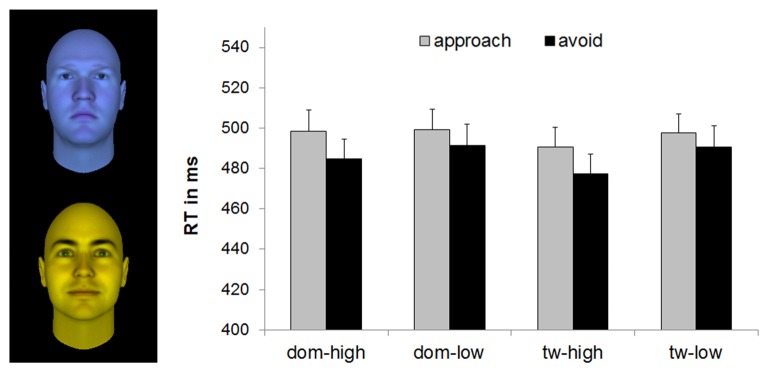
Mean reaction times (RTs; with standard errors) of the implicit joystick task for faces varying in dominance and trustworthiness, based on *n* = 49. Example faces of high dominance (upper face) and high trustworthiness (lower face) illustrate the blue and yellow filters applied to the stimuli from Oosterhof and Todorov (2008). Means were significantly different at *p* < 0.05 for avoidance vs. approach responses, for high vs. low features, and for high vs. low trustworthy faces. dom, dominance; tw, trustworthiness.

For the explicit joystick task and the rating task, stimuli were presented in their natural coloring, whereas for the implicit joystick task, pictures were presented with a blue or yellow filter (see Figure 1). The computer screen had a resolution of 1024 × 768 pixel. In the joystick tasks, initial picture size was 304 × 363 pixel, with full joystick displacement resulting in a picture size of 556 × 663 pixel for pulling, and a picture size of 112 × 134 pixel for pushing, respectively. For the rating, pictures were presented with a size of 332 × 396 pixel, via Presentation Software (Neurobehavioral Systems, Albany, CA, USA). A Logitech Attack 3 (Logitech, Newark, CA, USA) was used for responding in the joystick tasks.

### Tasks

#### Implicit Joystick Task

Each stimulus was presented twice, once with yellow and once with blue coloring, entailing a total number of 200 experimental trials, distributed across two blocks. Each trial was self-paced and started with a blank screen and the joystick in the resting (upward) position. To initiate stimulus presentation in the center of the screen, participants pressed the fire button. Participants were instructed to respond as fast as possible to the color of the face by pushing the joystick away from or pulling it toward their body. The joystick movement implied that participants’ arm moved likewise and caused the stimulus to shrink or grow in size. It disappeared when the minimum, respectively the maximum size was reached. During the eight practice trials, the stimulus remained visible after an erroneous response (allowing participants to practice until the response was correct). The stimulus-response mapping remained constant per participant, but was counterbalanced between participants, i.e., half of the participants reacted to yellow with pull movements and to blue with push movements, and the other half vice versa.

#### Explicit Joystick Task

The general setup was identical to the implicit joystick task, but instructions differed. Stimuli were presented in six blocks with 50 experimental trials each, preceded by eight practice trials each. In the first four blocks, stimuli were presented separately per feature, i.e., two blocks with faces varying in trustworthiness, and two blocks with faces varying in dominance. In these blocks, participants had to judge whether that particular feature was present, e.g., react to trustworthy faces with pull movements and to non-trustworthy faces with push movements. After the first block of each feature, the stimulus-response mapping was reversed (for this example, pull non-trustworthy faces and push trustworthy faces). Half of the participants started with evaluating trustworthiness, the other half started with evaluating dominance. The last two blocks combined both features and consisted of pictures corresponding to +2SD trustworthiness and +2SD dominance. Participants were instructed to react to one feature with pull movements, and to the other feature with push movements. This instruction was kept constant with the instruction in block #4, e.g., if participants were to pull trustworthy faces in block #4, they had to pull trustworthy faces and push dominant faces in block #5, which then reversed from block #5 to block #6. Taken together, this implies that participants had to differentiate either between high and low expressions of the same feature, or between high-trustworthy and high-dominant faces, but not between low-trustworthy and low-dominance faces within the same block.

#### Approach-Avoidance Rating

The same 100 stimuli as in the previous paradigms were presented each in the center of the screen with the rating scale below until a response was given. Participants should imagine standing face to face with the person depicted and explicitly rate their tendency to approach or avoid him as the number of steps they would make towards (+) or away (−) from him on a scale from −4 to +4. This rating scale was intended to capture conscious behavioral tendencies, in contrast to motor reactions as in the joystick tasks. As in prior studies (e.g., Radke et al., 2015a), the rating was always presented after the joystick paradigms to prevent carry-over effects from conscious evaluation of the pictures to behavioral tendencies.

### Endogenous Testosterone Concentrations

Saliva samples were taken using SaliCaps (IBL International, Hamburg, Germany) at the beginning of the experimental session. Samples were stored at −30°C until assessment by a commercial laboratory (ISD, Malente, Germany). Free active testosterone was determined via enzyme-linked immunosorbent assay (ELISA, Demeditec Diagnostics GmbH), based on the principle of competitive binding, and with a sensitivity of 2.2 pg/ml. Samples were analyzed in duplicate and the average was used in subsequent analyses. The intra-assay CV was 6.8%, and the inter-assay CV from this laboratory was below 10%. Testosterone levels could not be determined for one participant, and another participant showed high levels of >400 pg/ml. These two participants were excluded from the correlational analyses involving salivary testosterone.

### Digit Ratio (2D:4D)

Testosterone’s early organizational effects were indexed by participants’ digit ratio (Zheng and Cohn, 2011). For that purpose, participants’ left and right hands were photocopied. On these photocopies, the length of the second (2D) and the fourth (4D) fingers were measured from tip to basal crease later by two independent investigators. As the reliability between the two judgments was high (*α* > 0.98 for all measures), the mean of the two measurements was used for subsequent analyses, i.e., calculating the 2D:4D. The 2D:4D of the left and the right hand were significantly correlated, *r* = 0.546, *p* < 0.001.

### Approach-Avoidance-Related Traits

All participants completed the German version of the BIS-BAS scale (Action Regulating Emotion Systems Scale; ARES; Hartig and Moosbrugger, 2003), which assesses BIS-sensitivity with the subscales *anxiety* and *frustration* and BAS-sensitivity with the subscales *drive* and *gratification*. Additional demographic data was surveyed, e.g., education and relationship status, and is presented in Table 1.

**Table 1:** Characteristics of study participants (presented as Mean [SD] or *n*, of *n* = 50).

Age	25.10 (3.87)
Salivary testosterone (pg/ml; *n* = 48)	94.84 (49.02)
2D:4D left hand	0.96 (0.03)
2D:4D right hand	0.97 (0.04)
BIS	19.48 (4.09)
BAS	34.08 (3.14)
Sexual orientation (heterosexual/homosexual)	49/1
Relationship status (single/in a relationship/married)	23/25/2
Highest education (university degree/High School, i.e., “Abitur”/lower)	4/45/1
Current occupation (student/doctor/post-graduate/other)	42/3/2/3

*Note: BIS, Behavioral Inhibition System; BAS, Behavioral Approach System*.

### Statistical Analyses

For the joystick tasks, reaction time (RT) was defined from stimulus onset until movement onset. Trials with incorrect and extreme responses (>3SDs of the subject-specific mean per condition) were excluded [implicit: 5.5%, explicit: 20%]. For each joystick task, mean RTs were calculated for each level of the three experimental factors (Feature, Level, Movement). Participants with <5 operational trials per condition were excluded, yielding data of 49 participants for the implicit joystick task, and data of 44 participants for the explicit joystick task. Mean RTs were subjected to two repeated-measures ANOVAs with the within-subject factors Feature (trustworthiness, dominance), Level (high, i.e., +2SD, low, i.e., −2SD) and Movement (approach, avoid). Analogously, error rates were analyzed by using two repeated-measures ANOVAs with the within-subject factors Feature (trustworthiness, dominance), Level (high, i.e., +2SD, low, i.e., −2SD) and Movement (approach, avoid).

In addition, means of the rating scores per Feature and Level were analyzed using a repeated-measures ANOVA with the within-subject factors Feature (trustworthiness, dominance) and Level (high, i.e., +2SD, low, i.e., −2SD).

Following previous studies with emotional expressions (e.g., Radke et al., 2013, 2014), individual behavioral tendencies were calculated by subtracting mean RTs for pull movements from individual mean RTs for push movements. Here, positive scores reflect a relative approach tendency, while negative scores denote an avoidance tendency. These tendencies were used for computing Pearson’s correlations: (i) between tasks; and (ii) with indicators of endogenous and prenatal testosterone as well as trait approach-avoidance motivation. Statistical testing was performed with IBM SPSS 22.0 with an α-level of *p* < 0.05 and partial eta squared as estimate of effect size.

## Results

### Implicit Joystick Task

The Feature × Level × Movement ANOVA on the RTs showed a significant main effect of Movement, *F*_(1,48)_ = 7.08, *p* = 0.011, partial *η*^2^ = 0.13, a significant main effect of Level, *F*_(1,48)_ = 10.88, *p* = 0.002, partial *η*^2^ = 0.19, and a significant Feature × Level interaction, *F*_(1,49)_ = 4.29, *p* = 0.044, partial *η*^2^ = 0.08. No other effects were significant, *F*s < 2.49, *p*s > 0.12.

The main effect of Movement was due to faster avoidance (*M* = 485.91 ms, *SD* = 67.36) than approach (*M* = 496.44 ms, *SD* = 66.70) reactions. The main effect of Level was due to faster reactions for the faces indicating the presence of a particular feature, i.e., high-trustworthy and high-dominant faces (*M*_high_ = 487.67 ms, *SD* = 65.59, compared to low-trustworthy and low-dominant, *M*_low_ = 494.68 ms, *SD* = 66.41). This effect was driven by the faces varying in trustworthiness as decomposing the Feature × Level interaction revealed faster reactions for faces signaling high, compared to low, trustworthiness, *F*_(1,48)_ = 18.84, *p* < 0.001, partial *η*^2^ = 0.28, without significant differences in RTs to faces varying in dominance, *F*_(1,48)_ = 1.73, *p* = 0.19, partial *η*^2^ = 0.04 (see Table 2 for means and Figure 1).

**Table 2:** Performance of study participants in the two joystick tasks in ms (presented as Mean [SD]).

	Implicit (*n* = 49)		Explicit (*n* = 44)	
	Approach	Avoid	Approach	Avoid
**Trustworthiness**				
High	490.4 (70.5)	477.3 (68.1)	1026.1 (238.2)	984.0 (228.8)
Low	497.7 (66.2)	490.4 (74.6)	1065.6 (319.5)	1012.0 (361.6)
**Dominance**				
High	498.5 (73.0)	484.6 (70.2)	935.7 (265.6)	935.2 (249.2)
Low	499.2 (71.4)	491.4 (73.1)	997.9 (239.0)	997.0 (237.2)

The Feature × Level × Movement ANOVA on the error rates showed no significant effects, *Fs* < 3.82, *p*s > 0.06 (see Table 3 for means).

**Table 3:** Performance of study participants in the two joystick tasks in error rates (presented as Mean [range]).

	Implicit (*n* = 49)		Explicit (*n* = 44)	
	Approach	Avoid	Approach	Avoid
**Trustworthiness**				
High	3.2 (0–16)	4.3 (0–28)	21.2 (2–56)	18.5 (0–62)
Low	4.5 (0–16)	4.8 (0–28)	18.5 (0–76)	17.3 (0–56)
**Dominance**				
High	2.9 (0–16)	4.2 (0–20)	20.6 (2–54)	14.7 (2–44)
Low	4.2 (0–16)	3.3 (0–20)	17.3 (0–72)	18.3 (0–72)

### Explicit Joystick Task

The Feature × Level × Movement ANOVA on the RTs showed significant main effects of Movement, *F*_(1,43)_ = 4.41, *p* = 0.042, partial *η*^2^ = 0.09, of Level, *F*_(1,43)_ = 4.76, *p* = 0.035, partial *η*^2^ = 0.10, and of Feature, *F*_(1,43)_ = 9.81, *p* = 0.003, partial *η*^2^ = 0.19. There was also a significant Feature × Movement interaction, *F*_(1,43)_ = 4.15, *p* = 0.048, partial *η*^2^ = 0.09. No other effects were significant, *F*s < 0.41, *p*s > 0.53.

**Figure 2:**
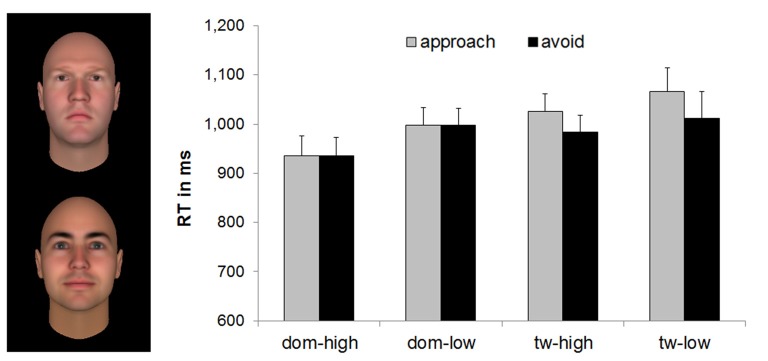
Mean RTs (with standard errors) of the explicit joystick task for faces varying in dominance and trustworthiness, based on *n* = 44. Example stimuli show high dominance (upper face) and high trustworthiness (lower face) from Oosterhof and Todorov (2008). Means were significantly different at *p* < 0.05 for avoidance vs. approach responses, for high vs. low features, for faces varying in dominance vs. faces varying in trustworthiness, and for avoidance vs. approach to faces varying in trustworthiness. dom, dominance; tw, trustworthiness.

As in the implicit task, the main effect of Movement was due to faster avoidance (*M* = 982.08 ms, *SD* = 231.85) than approach (*M* = 1006.29 ms, *SD* = 235.28) reactions. Similarly, the main effect of Level was due to faster reactions for the faces indicating a high level of trustworthiness or dominance (*M*_high_ = 970.25 ms, *SD* = 227.23, compared to low-trustworthy and low-dominant faces, *M*_low_ = 1018.12 ms, *SD* = 255.22). The main effect of Feature was evident in faster reactions to faces varying in dominance (*M* = 966.45 ms, *SD* = 216.54) than to faces varying in trustworthiness (*M* = 1021.93 ms, *SD* = 257.30). Follow-up analyses of the Feature × Movement interaction indicated that the effect of Movement, i.e., faster avoidance than approach RTs, was only present for faces varying in trustworthiness, *F*_(1,43)_ = 7.17, *p* = 0.01, partial *η*^2^ = 0.14, but not for those varying in dominance, *F*_(1,43)_ < 0.01, *p* = 0.97, partial *η*^2^ < 0.01 (see Table 2 for means and Figure 2).

For the error rates, the Feature × Level × Movement ANOVA showed a significant main effect of Movement, *F*_(1,43)_ = 8.46, *p* = 0.006, partial *η*^2^ = 0.16. No other effects were significant, *Fs* < 3.43, *p*s > 0.07. More errors were committed when approach movements were required (*M* = 19.40%, *SD* = 15.75) than when avoidance movements were required (*M* = 17.18%, *SD* = 14.23; see also Table 3).

### Approach-Avoidance Rating

The Feature × Level ANOVA on the rating showed a significant main effect of Feature, *F*_(1,49)_ = 144.95, *p* < 0.001, partial *η*^2^ = 0.75, and a significant Feature × Level interaction, *F*_(1,49)_ = 181.89, *p* < 0.001, partial *η*^2^ = 0.79. The main effect of Level was not significant, *F* = 0.33, *p* = 0.57.

**Figure 3:**
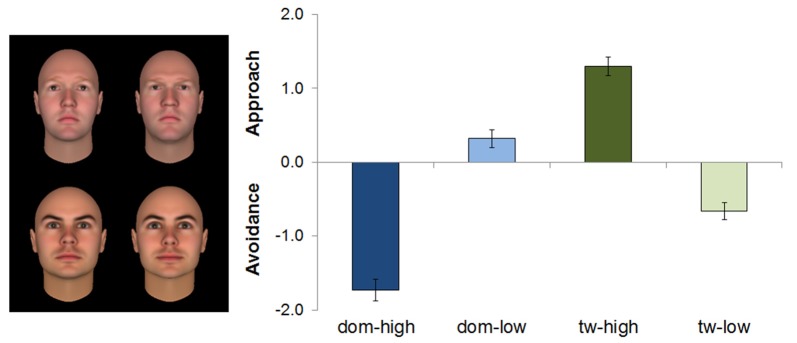
Mean ratings (with standard errors) of the rating task for faces varying in dominance (upper row: left: low, right: high) and trustworthiness (lower row: left: low, right: high), as derived from the Feature × Level interaction, *n* = 50. Positive scores indicate approach, whereas negative scores indicate avoidance tendencies. All means differ significantly from zero and from each other at *p* < 0.05. dom, dominance; tw, trustworthiness.

The main effect of Feature was due to higher ratings for the faces varying in trustworthiness than for the faces varying in dominance. This effect, however, needs to be viewed in the context of the Feature × Level interaction, which revealed differences between “high” and “low” versions for both features, yet in opposite directions. In other words, for faces varying in trustworthiness, highly trustworthy faces were rated as more approachable (*M* = 1.30, *SD* = 0.90) than faces signaling low trustworthiness (*M* = −0.66, *SD* = 0.80). For faces varying in dominance, low-dominant faces received higher approach ratings (*M* = 0.32, *SD* = 0.85) than highly dominant faces (*M* = −1.73, *SD* = 1.06; see also Figure 3).

### Correlations

First, there were no significant correlations between behavioral tendencies in the joystick tasks and the rating (all *p*s > 0.13). Secondly, there were no significant correlations between indicators of testosterone and approach-avoidance tendencies in any of the three tasks. The lowest *p*-value was *p* = 0.052, obtained for the (negative) association between salivary testosterone and the approach tendency toward low-trustworthy faces in the explicit joystick task (*r* = −0.28). Endogenous and prenatal indicators of testosterone were not significantly correlated with one another, *r* = −0.23 (*p* = 0.12), and *r* = −0.17 (*p* = 0.26) for left and right hand respectively, nor with trait approach-avoidance motivation (all *p*s > 0.18). Trait approach-avoidance motivation was not significantly correlated with motivational tendencies (all *p*s > 0.08).

## Discussion

The current study investigated three “levels” of approach-avoidance tendencies to faces varying in the social traits of trustworthiness and dominance. When participants explicitly rated their behavioral tendencies, highly trustworthy faces elicited approach, while untrustworthy faces elicited avoidance. Conversely, highly dominant faces yielded behavioral avoidance, whereas faces signaling low dominance yielded approach. This pattern, however, did not manifest in the joystick tasks where action tendencies were assessed via the initiation of approach-avoidance movements. Instead, more general effects of faster avoidance movements and faster reactions to faces signaling high trustworthiness or high dominance emerged. Neither endogenous nor prenatal indicators of testosterone were related to motivational tendencies.

The two dimensions proposed by Oosterhof and Todorov (2008) can provide a useful framework for investigating motivational tendencies to social traits. As derived from their model of face evaluation, our rating data underpinned that facial variations in trustworthiness and dominance influence approach-avoidance decisions. High trustworthiness and low dominance prompted approach, and low trustworthiness and high dominance prompted avoidance. Of these, faces indicating the presence of a particular feature, i.e., high-trustworthy and high-dominant, evoked the strongest tendencies. As for emotional expressions, recognition of these “present” features may be easier because they lie closer to the prototypical expressions (Young et al., 1997), thereby reducing ambiguity. Moreover, the mechanisms and neural circuits involved in evaluating emotional expressions also take effect in the judgment of social traits, and judgments are often highly correlated (Todorov and Engell, 2008; Engell et al., 2010). However, we did not assess judgments on these social traits, but probed participants for the behavioral consequences.

In contrast, behavior in the joystick tasks did not manifest as approach-avoidance tendencies, i.e., faster approach toward one type of stimuli and faster avoidance toward another, as commonly observed for emotional faces (Rotteveel and Phaf, 2004; Phaf et al., 2014). Comparing not the two movement directions, but trustworthy and untrustworthy faces, Slepian et al. (2012) reported an asymmetry with trustworthy faces eliciting faster approach than untrustworthy faces, without differences in avoidance. In fact, our data also reveal faster approach movements for high than for low trustworthy faces. However, this effect was neither limited to approach nor reversed for avoidance movements, i.e., there was also faster avoidance of high vs. low trustworthy faces. Therefore, this partial replication of Slepian et al. (2012) needs to be interpreted with caution as it is likely overshadowed by general effects, e.g., of movement. Accordingly, the overall faster initiation of avoidance than approach movements in the current study was unexpected based on previous studies using the same response measures and setup with emotional faces (e.g., Radke et al., 2013, 2014). Taken together, this limits the interpretation of the findings in terms of behavioral *tendencies*, i.e., RT differences, instead of general responses to faces varying in social traits.

The findings from the joystick tasks may also indicate that the judgment of social traits does not map as readily on approach-avoidance tendencies as evaluations of emotional valence. The stimulus set of the current study was drawn from the range of faces rated as emotionally neutral (Oosterhof and Todorov, 2008) in order to exclude this potential affective confound. One may speculate whether more extreme facial displays of social traits would evoke distinct behavioral tendencies in the joystick tasks, but disentangling valence judgments from judgments of trustworthiness and dominance is inherently difficult at the extremes of the two dimensions. Moreover, it seems plausible that emotional expressions, being dynamic, demand faster action and more distinct behavioral tendencies than less transient social signals. In light of the unexpected overall faster avoidance responses, using context-deprived computerized faces may also warrant further investigation. The computerized faces from this database have been used in a variety of studies investigating social perception, such as when selecting allies for a team (Kret and De Dreu, 2013) and in automatic threat processing (Shasteen et al., 2015). Still, they might be perceived as artificial, especially when combined with a colored filter, and they might be less familiar than natural faces, possibly leading to negative evaluations due to difficulties in extracting the relevant diagnostic information (Winkielman et al., 2003).

Ascending “levels” of approach-avoidance tendencies were to be assessed by instructions that varied in the explicitness of the evaluation, i.e., from reacting to the irrelevant characteristic of color, to judging the facial traits, to indicating one’s behavioral tendency. In line with previous research (Phaf et al., 2014; Radke et al., 2015a), approach-avoidance tendencies were evident for the most explicit instructions yielding conscious evaluation of one’s behavior. Yet, no tendencies emerged for either variant of the joystick task. Instead, faster reactions to high-trustworthy faces were evident in the implicit task version, whereas in the explicit task, it was faces varying in dominance that elicited the fastest responses. Facial trustworthiness is neurally evaluated without perceptual awareness (Freeman et al., 2014; Marzi et al., 2014), whereas intentional trustworthiness judgments are driven by a different neural circuitry (Winston et al., 2002). As there are no analogous studies on dominance perception which would point to a similar precedence in neural processing, trustworthiness might more easily influence behavior particularly when attention is directed toward other stimulus features (i.e., color in our task). Interestingly, however, dominance perception is influenced by contextual cues, with masculinized distractor faces decreasing perceived dominance of the target face (Re et al., 2014). Such a relative evaluation of dominance may facilitate more deliberative judgments, as required in our explicit joystick task. Yet, these dominance evaluations might not only be affected by the faces shown within the same block, but also by participants’ perception of their own dominance and their interpretation of “dominance”, which we did not assess or specify in terms of physical or social dominance (see also Re et al., 2014). Taken together, these results may suggest that trustworthiness and dominance might be differentially processed in implicit and explicit contexts.

Unlike prior studies that relied on affect and gender evaluations as explicit and implicit evaluations, respectively (Roelofs et al., 2009), the two currently used instructions turned out not to be matched for difficulty. Of these two, the explicit joystick task prompted longer RTs and higher error rates, which was underlined by participants’ spontaneous accounts of increased task difficulty and confusion. Moreover, handling a more stringent cutoff (e.g., at least 70% valid trials per condition) would have drastically diminished the sample size for analyses in the explicit joystick task. We acknowledge that this unexpectedly high task difficulty may limit the interpretation of the results from this task. Still, the pattern of error rates matches that of RTs, with overall more errors and more hesitation when approach movements were required, suggesting that approach movements were particularly difficult in this task. In part, this may reflect the increased processing demands associated with evaluating faces in terms of trustworthiness or dominance compared to following an arbitrary response mapping. Adding difficulty, the stimulus-response mapping changed after each block of the explicit joystick task, but remained constant throughout the implicit version. To move toward matching task demands, future studies should consider using a block-wise changing of the mapping in both tasks.

Contrary to our hypotheses, testosterone was not associated with approach tendencies to faces signaling threat via the intention or the ability to cause harm, i.e., untrustworthy or dominant faces. Previous research indicates that testosterone can alter the perception of trustworthiness (Bos et al., 2010; Carré et al., 2014) and dominance (Welling et al., 2016; but see Bird et al., 2017) as well as induce threat approach (Enter et al., 2014, 2016). Despite the null effect in the current study, the possibility still exists that heightened testosterone went hand in hand with a decreased sensitivity to cues of trustworthiness or dominance, without translating into behavioral changes. Given the absence of approach-avoidance tendencies in the joystick tasks, the lack of associations with testosterone may not be surprising. However, testosterone did not influence explicit approach-avoidance ratings either, although distinct motivational patterns were evident on this level. Together, these findings could point to the fact that variations in endogenous testosterone, on their own, may not be sufficient to explain variations in motivational behavior, and may need stronger contextual triggering or further regard of individual differences, e.g., trait dominance or cortisol (Mehta and Josephs, 2010; Carré and Mehta, 2011; Carré et al., 2017).

Another consideration is that many of the above mentioned findings were obtained in the context of testosterone administration leading to supraphysiological levels (in females: Bos et al., 2010; Enter et al., 2014, 2016), while our design was correlational and included only one measure of salivary testosterone in its normal range. Fluctuations in testosterone may be more relevant than baseline levels for modulating social motivational behavior such as aggression (Carré et al., 2009b, 2013, 2014). In a similar vein, prenatal testosterone seems to play a stronger role in activational effects of the hormone for higher order social cognition than for basic reactions to threat (Terburg and van Honk, 2013; Terburg et al., 2016). However, including only male participants certainly prevents generalization across sexes.

Nevertheless, the current study helps clarify the hypothesized link between social traits and approach-avoidance responses by testing the proposition of Oosterhof and Todorov (2008). By showing that trustworthiness prompted approach ratings and dominance prompted avoidance ratings, without similar behavioral responses, our findings add to a body of research on approach-avoidance tendencies to socially relevant stimuli. They also contribute to our understanding of the impact of hormones on social motivation. The current null-relation between testosterone and motivational tendencies may point to specific contextual boundaries under which effects are (not) to be expected that need to be investigated further. Together with the inconsistencies of past literature and the limitations of the present study, future research is needed in order to replicate and expand upon the current findings.

## Author Contributions

SR and BD designed the study. TK collected and processed the data as part of her dissertation. SR performed data analyses of the joystick tasks with support from LW. TK performed data analyses of the rating task as part of her dissertation. SR drafted the manuscript. All authors revised the manuscript and gave final approval of the version to be published.

## Conflict of Interest Statement

The authors declare that the research was conducted in the absence of any commercial or financial relationships that could be construed as a potential conflict of interest.
